# Design Mapping: A Conceptual Framework for Co‐Designing Evidence‐Based Digital Mental Health Programs

**DOI:** 10.1111/hex.70385

**Published:** 2025-09-03

**Authors:** Kelsie Bufton, Maria Bates, Matthew Fuller‐Tyszkiewicz, Jasmin Hamid, Elizabeth Westrupp

**Affiliations:** ^1^ School of Psychology Deakin University Geelong Victoria Australia; ^2^ School of Communication and Creative Arts Deakin University Geelong Victoria Australia; ^3^ Independent Consultant Geelong Victoria Australia

**Keywords:** codesign, design thinking, healthcare, intervention, mental health

## Abstract

**Introduction:**

There is currently limited guidance on how to codesign digital mental health programs in collaboration with end‐users. As a result, common barriers often prevent codesign initiatives from achieving their full potential in enhancing the effectiveness of digital mental health supports. These barriers include the exclusion of end‐users from the early stages of design, a lack of attention to diversity in user experiences and needs, failure to use tools that facilitate creative and egalitarian collaboration, and prioritisation of usability over robust research methods. To address this gap, this article aims to present a novel conceptual framework, called ‘Design Mapping’, for developing digital mental health programs.

**Methods:**

The Design Mapping framework was developed through a three‐stage process. First, industry experts were consulted, and a review of relevant literature was conducted to identify current best practices for user collaboration in product/service development. Second, the initial conceptual development framework was created, drawing on the strengths of different approaches. Third, the framework was applied to develop a novel early childhood parenting program (called ‘Active Play’), enabling the approach to be tested and refined.

**Results:**

Design Mapping is a three‐phase framework that integrates tools from Design Thinking that emphasise meaningful and creative user collaboration, within a robust and systematic methodology inspired by Intervention Mapping. The framework also considers factors pertinent when developing both mental health and digital support programs. Preliminary findings from pilot testing of the Active Play program suggest that Design Thinking may support the development of programs that are feasible, effective and engaging.

**Conclusion:**

Design Mapping offers a novel conceptual codesign methodology for developing robust, evidence‐based support programs that are responsive to diverse user needs and preferences. The next step is to evaluate its effectiveness in comparison to existing methodologies.

**Public Contribution:**

This article presents Design Mapping, a novel conceptual framework that offers a systematic approach to developing evidence‐based digital mental health programs, emphasising meaningful and creative collaboration with end‐users throughout. The primary aim of this article is to explore and address current limitations in codesign practices in the healthcare industry, consider best practice for user collaboration in the development of digital mental health programs, and provide clear and pragmatic descriptions of suggested methods, with sufficient detail to support real‐world utilisation. The Design Mapping framework was conceptualised, tested and refined via the development of a novel early‐childhood parenting support smartphone‐app, called ‘Daily Growth’ (Figure 2). End‐users were involved throughout the development of Daily Growth, from initial program inception through to prototype testing and refinement. This article presents an overview of the development of Daily Growth to illustrate the application of Design Mapping, with other publications providing an in‐depth account of the methodology.

## 1 Introduction

Collaborating with target end‐users in mental health research has been shown to improve the relevancy, usability, effectiveness and engagement of supportive programs and resources [1, 2]. However, evidence‐based guidance on how to effectively codesign digital mental health programs with target end‐users remains limited [1]. To address this gap, we present ‘Design Mapping’, a novel conceptual framework for co‐designing content for digital mental health programs. Design Mapping integrates creative user collaboration tools from Design Thinking, a user‐centric innovation methodology increasingly applied in healthcare service design [3], with systematic and robust methodologies inspired by Intervention Mapping, a well‐regarded protocol for developing evidence‐based health programs [4]. By combining the strengths of these approaches, Design Mapping seeks to support the development of evidence‐based digital programs that address complex mental health problems and engage diverse end‐user groups.

To promote more meaningful, user‐oriented improvements in mental health outcomes, there is a growing global commitment to shift from expert‐led development work toward inclusive approaches that engage healthcare service users in the planning, design and evaluation of services [5, 6]. User‐centred development is particularly crucial when creating digital mental health programs. While human therapists can adapt in real‐time based on user responses, digital mental health programs must be pre‐adapted to accommodate a range of user needs and preferences [7, 8]. Despite this, end‐users are often excluded from the development of digital mental health programs, or their input is constrained by common barriers that limit its impact. First, effective codesign requires user involvement from the initial stages of development. However, many digital health studies engage users only during final usability testing to optimise technical features [1, 9], bypassing a cost‐effective opportunity to build solutions in alignment with user needs. Second, common user evaluation tools may only support superficial user input and reinforce the power dynamic of the researcher as ‘expert’. For example, standardised evaluation questionnaires are often constrained by the researchers' areas of interest and fail to capture aspects most important to the end‐user. This dynamic may inadvertently create power imbalances between researchers and participants that limit the free exchange of feedback and ideas and ultimately impede the creation of engaging and useful supports.

Third, traditional qualitative methods often lack tools that facilitate creative and collaborative idea generation between users, stakeholders and developers – processes essential to successful innovation [10, 11, 12]. While standardised research tools are essential for generating robust evidence of program effectiveness [3], it is equally important to use methods that engage end‐users in creative problem‐solving to ensure program concepts, content and designs reflect their needs and preferences.

Finally, codesign frameworks and digital health studies largely treat target‐users as a homogenous group [13], generalising findings from a subset of users and overlooking how to identify and address the distinct needs of underserved groups. Even when target users share common characteristics (e.g., mental health diagnoses, geographic location), their needs may differ based on factors, such as age, culture, level of need, values, beliefs and motivations. Without meaningful investigation of this diversity, the generalisability of codesign findings may remain limited [14]. While it may be infeasible for a single mental health program to fully address the needs and preferences of all user subgroups, usability guidelines suggest that designing for ‘extreme’ conditions and underrepresented groups can produce solutions that are useful to all [15, 16]. Therefore, treating users as a homogenous group and overlooking the needs of those missed by existing supports may limit the capacity of digital mental health programs to effectively engage diverse users at the population level [1, 9, 10, 17].

These barriers to effective codesign may partly stem from a lack of evidence‐based, practical guidance on how to meaningfully engage users and apply user‐driven insights during digital health innovation [18, 19]. Although the limitations of consumer collaboration in the sector are increasingly recognised [13], critiques of codesign studies often delineate idealised best practices without providing sufficient guidance for their systematic implementation [3]. We argue that this ambiguity impedes the ability of researchers to understand and implement codesign best practices in real‐world settings. Addressing these limitations may enhance the effectiveness of user collaboration and support digital programs in realising their full potential to widen the reach and engagement of evidence‐based mental health treatments [1, 17, 20].

### 1.1 The Current Study

This study aimed to develop a novel conceptual framework, Design Mapping, for developing digital mental health programs. The framework seeks to address existing limitations in codesign practices and enhance the quality and consistency of user collaboration initiatives in healthcare innovation. Design Mapping was developed through a three‐stage process based on established methods used in conceptual framework development in healthcare [21, 22, 23].

The first development stage involved consulting industry experts and reviewing relevant literature to identify and critically evaluate existing user‐centred approaches. In the second stage, the Design Mapping framework was conceptualized by integrating the strengths of existing methodologies. The final stage involved testing and iteratively refining the conceptual framework through the development of Daily Growth, a novel smartphone app designed to support parenting during early childhood (Figure 2). Daily Growth seeks to address previous barriers of population level digital parenting interventions by: (1) providing in‐the‐moment micro‐interventions (brief video resources) when parents and/or children experience emotion dysregulation; (2) offering parents three types of parenting support (Emotion Coaching; Active Play; Wayapa Wuurrk) to cater to different parent needs and (3) tailoring each video resource to specific parenting situations. All three parenting programs are focused on building parent and child emotion regulation to prevent child mental health problems. To illustrate the Design Mapping Framework, we will focus specifically on the development of the ‘Active Play’ program, which aims to support parents to use play‐based physical activity to manage difficult parenting situations.

## 2 Methods and Results

### 2.1 Stage 1: Assessing Existing Approaches

The first stage involved identifying and evaluating existing development methodologies to inform the creation of a novel approach for co‐designing digital mental health programs.

#### 2.1.1 Stage 1: Aims

1. Identify best practice in user‐centred design for product and service development, with a focus on addressing common limitations in codesign within healthcare.
2. Examine how these user‐centred methodologies are currently implemented in healthcare innovation and evaluate their effectiveness in addressing sector‐specific complexities.
3. Identify alternative development frameworks that offer strengths capable of addressing the limitations of user‐centred approaches.

#### 2.1.2 Stage 1: Method

A four‐part process was used to address these aims:

1. **Expect Consultation – Design:** Professionals from commercial and academic sectors, including experts in user experience (UX) research, interaction design and usability, were consulted. These experts provided practical insights into the strengths and limitations of existing user collaboration methods based on real‐world applications.
2. **Targeted literature review 1:** A focused review of relevant literature examined how user‐centred methodologies are applied in healthcare contexts. The search term included:
  - (‘Human‐centred design’ OR ‘design thinking’ OR ‘user‐centred design’ OR ‘user experience’) AND (‘healthcare’ OR ‘mental health’ OR ‘mental healthcare’).
3. **Expect Consultation – Public Health Frameworks:** Experts in public health intervention development were consulted to identify alternative frameworks that may effectively address the complexities of healthcare innovation.
4. **Targeted literature review 2:** A second targeted review of published literature evaluated the strengths and limitations of selected public health frameworks in the context of co‐designing digital mental health programs. Search terms included:
  a. ‘Intervention mapping’ AND (‘digital’ OR ‘mHealth’ OR ‘digital health’ OR ‘eHealth’ OR ‘telehealth’).
  b. ‘Intervention mapping’ AND ‘mental health’.

#### 2.1.3 Stage 1: Results

##### 2.1.3.1 Design Thinking

Consultation with industry experts identified Design Thinking as a user‐centred development approach with multiple strengths suited to addressing current limitations in healthcare codesign practices. Notably, the term ‘Design Thinking’ is often used interchangeably in the literature with other conceptually similar approaches, including Human‐Centred Design and User‐Centred Design. Consistent with a recent review of design methods in healthcare, this article uses Design Thinking as an umbrella term encompassing all three approaches [24].

Design Thinking is distinguished from traditional linear, top‐down approaches by prioritising usability above all else [24, 25]. It is an iterative, nonlinear process that prioritises creativity and emphasises building empathy for end‐users to develop solutions that meaningfully address the target problem [25, 26]. End‐users and stakeholders are engaged throughout all stages of development, from conception to implementation, with a focus on delineating power hierarchies to support equitable co‐creation [17]. Once a multi‐disciplinary team is established, Design Thinking comprises of three iterative, nonlinear phases: (1) ‘inspiration’‐ gaining a deep understanding of the problem and building empathy with end‐users; (2) ‘ideation’, creatively generating solutions and (3) ‘implementation’, developing a marketing strategy for the final product or service (see Supporting Material S1: Table 1 for details) [24, 26].

Design Thinking originated in the private sector, however is increasingly being adopted to address complex social issues. A systematic review identified 32 studies applying Design Thinking methods to health‐related innovation [3]. While empirical comparisons to other development methodologies are limited, emerging evidence suggests that Design Thinking produces interventions that have positive effects on health outcomes [3]. However, before conclusions can be drawn about the suitability of Design Thinking for healthcare innovation, higher‐quality empirical research is required [24]. This is complicated by inconsistencies in how Design Thinking is defined and executed, along with a lack of detailed methodological reporting in studies utilising the approach [3, 24]. Additionally, while several design agencies offer resources to guide the flexible application of the Design Thinking methodology, these can be difficult to apply within a systematic development approach without the right expertise [27, 28, 29]. Providing accessible frameworks for applying Design Thinking in health may aid the replication and empirical assessment of the methodology.

Design Thinking has also faced criticism for lacking methodological rigour and depth of insights compared to health research standards [3]. The fundamental tenets of Design Thinking, embracing ambiguity, generating innovative ideas, prioritising usability and using intuition to synthesise data [26, 28], conflict with many of the constraints of health research, which requires rigorous testing to ensure evidence‐based effectiveness [3, 24, 25]. Furthermore, research funding and ethics processes require predefined development, evaluation and implementation plans. These factors can impede early end‐user involvement, necessitate the use of robust research methods over intuition in data analysis, and require positive health outcomes to be prioritised over usability [19].

Additionally, Design Thinking tools may prove insufficient for addressing the complexity of digital health innovation [24]. For example, user personas are fictional archetypes used to illustrate user needs, experiences and behaviours [29]. Personas typically draw on demographic characteristics such as gender and personal circumstances such as living with disability. While such characteristics can be relevant, personas that focus on these attributes may be inadequate for developing complex health programs. This approach risks perpetuating unhelpful stereotypes and may obscure developers from uncovering deeper shared experiences, beliefs, values, preferences and barriers that fundamentally influence how healthcare innovation can be effective and engaging for diverse user groups [24, 30].

Given these limitations, ongoing debate among design researchers questions whether Design Thinking is compatible with scientific practice, suggesting it may need to be reconceptualised to meet the demands of health innovation. This has prompted calls for the establishment of a ‘design science’ comprising evidence‐based, systematic and formalised design methods grounded in the core values of empirical scientific research: objectivity, rationality and universalism [31]. This discourse highlights the potential value of a new intervention development framework that combines the creative strengths of Design Thinking with the rigour and complexity required in health research to support more meaningful and impactful user engagement in healthcare innovation.

##### 2.1.3.2 Intervention Mapping

Consultation with experts in public health intervention development identified Intervention Mapping as a well‐established, systematic methodology that may provide the rigour lacking in Design Thinking. Intervention Mapping supports the development of health promotion programs grounded in theoretical and empirical evidence, tested through rigorous data collection and analysis, tailored to the needs of target consumers, and capable of addressing complex, multi‐levelled, behaviour change needs [4, 32, 33]. The framework entails six iterative steps, beginning with a needs assessment to understand the health problem and ending with the development of an evaluation plan to assess program effectiveness (see Supporting Material S1: Table 1 for details) [4].

Intervention Mapping has been used to develop several digital health [34, 35] and mental health programs [36]. Although empirical comparisons with other development approaches are limited [32], evidence suggests that Intervention Mapping produces digital programs with positive effects on health‐related outcomes [37, 38]. While Intervention Mapping promotes community and stakeholder engagement, several limitations affect its practicality for co‐designing digital mental health programs. Its methodology is complex, time‐consuming and resource‐intensive, with limited flexibility to adapt to the varying needs, scopes and resources of different projects [39, 40]. Additionally, the framework lacks specific methods for creative, collaborative and egalitarian idea generation among users, stakeholders and developers – elements critical to successful innovation [10]. It provides limited guidance on how to foster a shared understanding of user needs within multidisciplinary development teams and on integrating user insights throughout program development [1, 24]. Finally, the framework largely overlooks strategies for addressing diverse end‐user needs, including those of underserved groups, which may limit its capacity to support inclusive, population‐level engagement [17].

This evaluation of Design Thinking and Intervention Mapping suggests that the strengths of each framework may help address the limitations of the other in developing digital health programs, providing an opportunity to create a new, integrated intervention development methodology.

### 2.2 Stage 2: Development of the Design Mapping Conceptual Framework

The second stage involved conceptualising the initial Design Mapping prototype, informed by findings from Stage 1.

#### 2.2.1 Stage 2: Aims

1. Integrate the complementary strengths of Design Thinking and Intervention Mapping into a prototype conceptual framework.
2. Incorporate considerations for digital content delivery.
3. Address challenges associated with designing services for individuals experiencing mental health difficulties.

#### 2.2.2 Stage 2: Method

To achieve these aims, a structured, multi‐method approach was used:

1. **Matrix Tool Development:** A matrix was created to systematically compare the key features, strengths and limitations of Design Thinking and Intervention Mapping. This analysis informed the initial Design Mapping prototype.
2. **Team Development Meetings**: The prototype was visually mapped and iteratively refined through meetings with professionals and academics in Design Thinking, digital health and intervention development. Discussions addressed factors relevant to digital program delivery and mental health service design, e.g., the limitations of traditional user personas in capturing the complexity of end‐user experiences in healthcare settings.
3. **Targeted Review of Literature:** A targeted review of relevant literature identified considerations for (a) designing engaging digital mental health programs and (b) addressing the needs of marginalised individuals experiencing mental health challenges. Search terms included:
  - (Engagement OR access OR feasible OR feasibility OR efficacy OR effectiveness) AND (‘digital mental health’ OR ‘digital health’ OR mHealth).
  - (Vulnerable OR marginalised OR underrepresented OR underserviced) AND ‘mental health’ AND (intervention OR program OR support).
4. **Prototype Development:** Insights from the literature review and team development meetings were synthesised and incorporated into the initial iteration of the conceptual Design Mapping framework.

#### 2.2.3 Stage 2: Results

**Figure 1:**
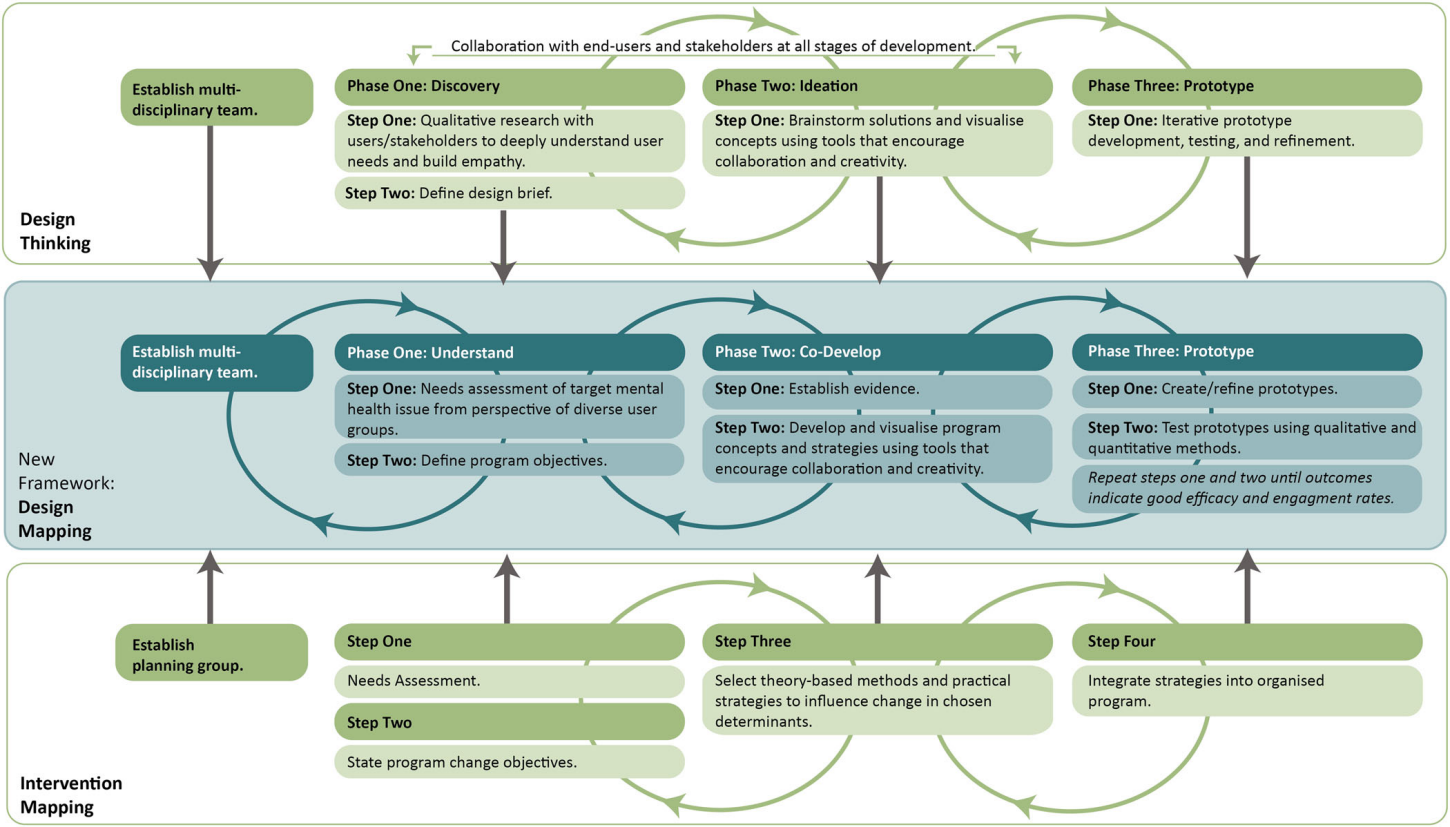
Design Mapping: A Framework for Developing Digital Mental Health Programs, Drawing Upon Design Thinking and Intervention Mapping. *Note:* Steps 5–6 of Intervention Mapping have been omitted, as they extend beyond the scope of the Design Mapping approach.

Table 1 (Supporting Material S1) highlights that, while Design Thinking and Intervention Mapping differ in underlying philosophy, strategic tools and operational techniques, they share several key commonalities in their development processes. This synergy informed the creation of a new conceptual framework, Design Mapping (Figure 1), for developing digital mental health programs.

The conceptual Design Mapping framework applies Design Thinking principles, including meaningful and creative end‐user collaboration, within a systematic development approach aligned with the rigour and complexity required in health research. The framework comprises three iterative phases, informed by the first two phases of Design Thinking and the first four steps of Intervention Mapping. The first, ‘Understand’, phase involves undertaking a Needs Assessment to understand the target mental health issue and the needs of those impacted. The second, ‘Codevelop’, phase focuses on collaboratively developing key program concepts and strategies with end‐users and stakeholders. The third, ‘Prototype’, phase involves iteratively creating, testing and refining prototypes of program components in collaboration with target users until outcomes indicate the program is likely to be effective and engaging. To support the utilisation of the Design Mapping approach, a step‐by‐step protocol is provided in Supporting Material S2 of this article.

The three Design Mapping phases support a mixed methods approach, combining qualitative techniques to understand user needs and codesign solutions, with quantitative techniques to test resources in broader samples [25]. Consistent with Design Thinking [27], and, to some extent, Intervention Mapping [4], the phases are designed to be flexible and nonlinear, enabling iteration as new insights emerge. For example, program objectives may be refined based on user insights uncovered during prototyping. While programs should ultimately be grounded in empirical evidence, developers are encouraged to embrace ambiguity as part of a process that favours learning from end‐users over relying solely on researcher expertise [26].

Design Mapping also supports the identification and inclusion of diverse and underserved user groups. While the framework recommends user input at various stages of content development, there is no prescribed amount. Rather than regarding user input as a ‘tick‐box exercise’, developers are encouraged to view it as an opportunity to enhance program outcomes. Ideally, user research should occur whenever existing data cannot answer questions about user needs, though in practice, user input will depend on available resources and project requirements [3]. Design Mapping also addresses key considerations for delivering support programs digitally (e.g., enhancing user engagement through personalisation features) [41]; as well as designing services for people experiencing mental health difficulties (e.g., addressing power dimensions within codesign processes to support equitable collaboration and idea sharing) [17, 42].

Design Mapping is intended to be accessible for researchers new to codesign and flexible to the scope, needs and resources of each project. While the framework considers factors relevant to designing program content for digital platforms, it does not guide the technical development or testing of the program, which requires software engineering expertise. Ultimately, the framework aims to improve the quality of user collaboration in digital health, supporting the development of engaging, effective and evidence‐based programs.

### 2.3 Stage 3: Testing and Refining the Design Mapping Framework

The conceptual Design Mapping framework was tested and refined through its application in developing content for the Active Play program. This section focuses on elements of the development process that illustrate how Design Mapping was applied and refined. Full details of the Active Play program's development are presented in separate publications [43, 44, 45].

#### 2.3.1 Stage 3: Aims

- Test and refine the initial Design Mapping prototype.
- Develop a revised prototype that integrates solutions to issues identified during the development of Active Play.

#### 2.3.2 Stage 3: Method

**Figure 2:**
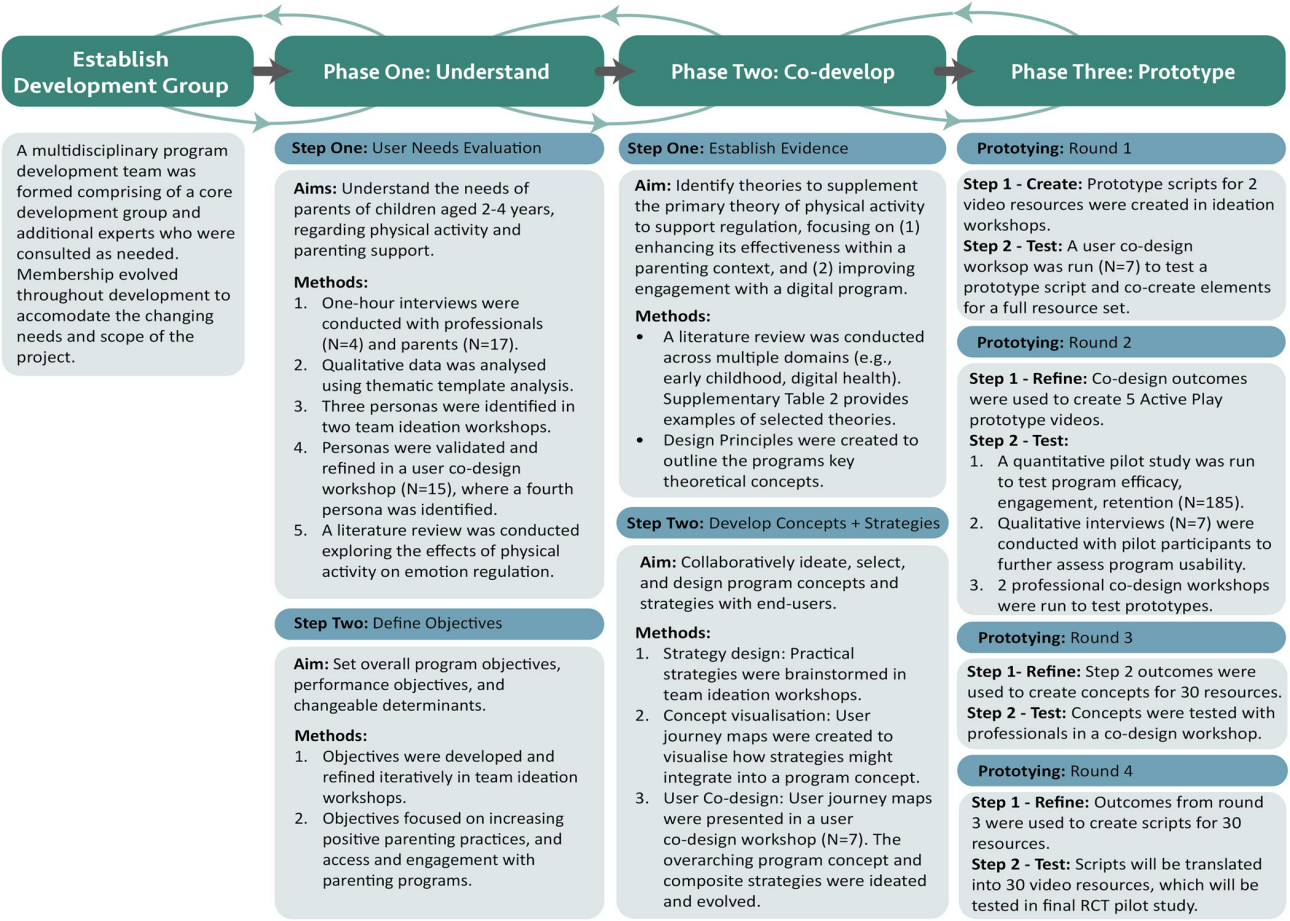
Application of Design Mapping to Develop the Active Play Program. *Note:* For examples of performance objectives, determinants, theories, practical strategies and codesign adjustments (see Supporting Material S1: Table 2).

1. **Testing the Design Mapping Framework**. Figure 2 outlines how the initial Design Mapping prototype was applied to develop content for the Active Play program and to test an early prototype of the Daily Growth app. A brief overview of each phase is provided below, with further details on specific tools available in the Design Mapping protocol (see Supporting Material S2). A comprehensive account of the codesign methodology used to develop the Active Play program is reported in separate publications [43, 44, 45].

##### 2.3.2.1 Phase 1: Understand

The first step of the ‘Understand’ phase involved a needs assessment to evaluate the nature and impact of emotion dysregulation in children aged 2–4 years, as well as the needs and experiences of parents in this domain. This assessment included a review of relevant literature and qualitative interviews with parents of young children (*n* = 17) and relevant professionals (*n* = 4). Interview data were analysed using thematic template analysis to identify overarching themes in user experiences [44, 46], with a set of prototype user personas developed to reflect diversity within these themes [43]. Both thematic findings and personas are presented in other publications [43, 44].

In the second step of Phase 1, a series of ideation workshops were run to identify program objectives and the factors needing to change to achieve these objectives (e.g., increase positive parenting practices by increasing parent knowledge of strategies that support child regulation). Ideation workshops bring together stakeholders and members of the program development team to creatively work with research insights and generate a diverse range of potential solutions to key design challenges [29].

##### 2.3.2.2 Phase 2: Codevelop

In Phase 2 (codevelop), Step 1 (Establish Evidence), a literature review was conducted to identify theoretical models to influence change in the selected determinants of the health‐promoting behaviour, e.g., parent knowledge of effective ways to respond to child dysregulation. In Active Play, physical activity was pre‐identified as a key theoretical change model (i.e., using aerobic activity to regulate the stress response), however additional models were selected to enhance program effectiveness and engagement. Table 2 (Supporting Material S1) provides further examples of selected theoretical models.

In Phase 2 (codevelop), Step 2 (Develop Program Concepts and Strategies), a set of design principles was developed to capture the projects key theoretical concept, give integrity and form to the program and ensure that key principles were preserved as the program evolved [28]. Practical strategies for implementing chosen theoretical methods were then brainstormed in team ideation workshops – see Supporting Material S1: Table 1 [4]. To visualise how these strategies might be experienced by users, journey maps were created to illustrate the user's interaction with the Active Play program from start to finish. These maps were refined in collaboration with parent end users during a codesign workshop. codesign workshops bring together users, stakeholders and members of the design team to collaboratively generate solutions to a shared problem [29].

##### 2.3.2.3 Phase 3: Prototype

In Phase 3, four iterative rounds of prototyping creation, testing and refinement were conducted. The first round involved creating low‐fidelity prototypes in the form of draft scripts for two video resources, which were tested and refined in collaboration with end‐users in a second codesign workshop. Insights from this workshop informed the development of five prototype video resources, which were tested through a pilot study, qualitative interviews and two codesign workshops with professionals. The outcomes of this testing guided the creation of concepts for the program's full set of 30 videos, which were then reviewed in a third codesign workshop with professionals. Final video resources were developed based on this feedback and all preceding stages and will be evaluated in a future randomised controlled trial of the Daily Growth program.

2. **Refining the Framework**. Throughout testing, the benefits and limitations of applying the framework's principles and methods to the development of the Active Play program were systematically documented and discussed in regular team meetings. Potential solutions to these limitations were explored through expert consultation and/or further review of relevant literature. These insights informed the third iteration of the conceptual Design Mapping protocol, which is included in Supporting Material S2.

#### 2.3.3 Stage 3: Results

This section outlines the key benefits observed of applying the Design Mapping framework in the development of the Active Play program, as well as its limitations and the measures taken to address them.

### 2.4 Advantages of the Design Mapping Framework

#### 2.4.1 Flexibility

The flexibility of the Design Mapping framework supported the development of the Active Play program. Several elements of the project were pre‐defined based on prior research by the development team. For example, program recipients were pre‐identified as parents of children aged 2–4 years, and physical activity was pre‐identified as a theoretical model for influencing positive change in emotion dysregulation [47, 48]. While these pre‐set parameters limited the extent to which user input could inform the setting of program objectives during Phase 1 (Understand), the needs assessment explored how digital parenting resources might be effective, feasible and engaging for diverse parents [44]. Insights from this assessment were applied throughout all stages of development. For example, many parents, including those from underserviced groups (e.g., LGBTQIA+ parents), expressed a fear of negative judgement when seeking professional parenting support. This finding informed several design choices aimed at enhancing inclusivity and representation, such as including parent testimonials to normalise parenting challenges and representing diverse families in resources.

#### 2.4.2 Iterative Approach

Adopting Design Thinking's iterative approach empowered the program development team to experiment with, test, and refine project components without the pressure of identifying optimal solutions from the offset. Consequently, the user personas, practical strategies, program concepts and design principles established during Phases 1 (Understand) and 2 (Codevelop) were iteratively updated as new insights emerged, resulting in a more nuanced and multi‐faceted representation of user needs and experiences [43]. We note drawbacks of this approach, including the additional time and steps added to the development process (e.g., piloting the prototype resources multiple times after modifications based on user feedback).

In Phase 3 (Prototype), iterative prototyping was embraced as an efficient way to enhance program efficacy and engagement. Using low‐fidelity prototypes made it feasible to complete four rounds of prototype creation and testing. Initial script testing in codesign workshops with end‐users proved cost‐effective and efficient. While producing the second set of prototypes (3‐min videos) was more resource‐intensive, their close resemblance to the final resources in structure and form enabled multiple rounds of feedback from users and experts across disciplines on key components, such as content, design, animation style [43]. This iterative process improved development efficiency by enabling potential barriers to efficacy and engagement to be identified and addressed before the costly production of the final program.

The first pilot study of the Active Play resources, reported in more detail in other publications, had promising results for feasibility, acceptability and preliminary efficacy [45]. Parents found the resources were useful, easy to use and relevant to their needs. Preliminary efficacy outcomes indicated Daily Growth's potential to improve parent emotion regulation and mental health. While retention rates were high, engagement with pre‐ and post‐surveys was lower than anticipated and varied by demographic group [45]. These findings informed further refinement and testing through additional qualitative and quantitative research with parent end‐users, stakeholders and experts [43, 44].

#### 2.4.3 Creative Tools for Complex Projects

In Phase 1 (Understand), user personas were built iteratively through collaborative brainstorming sessions involving the development team and end‐users. These exercises drew on themes and research insights obtained from standardised qualitative data analysis techniques [46]. By translating complex user data into tangible profiles, personas provided a visual representation of diverse user experiences grounded in empirical evidence and informed by multiple perspectives. Personas were employed throughout program development to align multidisciplinary decisions with user needs and values.

The persona approach was adapted to reflect the complexities of the Active Play project [24]. In response to user feedback, personas were framed not as fixed categories but as fluid mindsets and circumstances that parents might move between over time. Rather than building personas based on stereotypes of groups commonly missed by parenting supports (e.g., single parents), the focussed shifted to representing characteristics and circumstances that may increase the risk of disengagement. This approach aimed to represent the spectrum of individual experiences across and within demographic groups and to address underlying causes of engagement barriers [49].

In Phase 2 (codevelop), user journey maps were used to visualise how practical strategies could be integrated into a cohesive program concept. These maps were refined until the Active Play program concept was defined as a set of games tailored to address specific difficult parenting situations, each following a consistent format to offer various practical strategies. Journey maps also provided an efficient tool for testing the program concept with users in codesign workshops. Presenting users with three journey maps of different Active Play activities helped identify potential implementation barriers and supported collaborative problem‐solving. Users also reflected on how each journey might be adapted to better meet the needs of different personas.

#### 2.4.4 Systematic Development Approach

The conceptual Design Mapping framework appeared to provide a systematic approach that helped streamline a complex, multi‐component development process. It offered tools to meaningfully integrate user insights, support creative and collaborative ideation and ensure program components were grounded in empirical evidence. For example, in Phase 2 (codevelop), the selection of theoretical methods played a key role in anchoring program strategies in evidence. This step helped define the scope of user/stakeholder input in subsequent stages and guided the inclusion of user‐identified strategies based on their alignment with selected theories. Translating the selected theoretical models into design principles also offered a clear and accessible way to convey the project's core philosophies and values across the broader development team and process.

### 2.5 Framework Limitations

Several challenges emerged during the application of the Design Mapping framework, which were addressed in the protocol provided in Supporting Material S2. Initially, a diverse and representative sample of parent users was recruited via social media to participate in codesign activities. However, as the project progressed, attrition increased and maintaining diversity in participant recruitment became more difficult [43, 44]. In response, the protocol was updated to recommend forming community partnerships and design reference groups earlier in the process, and using snowball sampling to support ongoing recruitment of diverse user groups [50].

Additionally, the parent qualitative interviews conducted during the needs assessment were broad in scope and lengthy, making transcription, coding and analysis time‐consuming and resource‐intensive [44]. For projects with limited budgets and resources, the protocol recommends focusing qualitative enquiry on a smaller set of well‐defined research questions to more efficiently understand user needs and experiences.

Finally, the Active Play project did not evaluate the acceptability, feasibility or perceived value of the codesign process itself. Prior codesign studies in mental health have found that while some participants describe the process as ‘rewarding’ and ‘skill‐building’, others report it as ‘exhausting’ and ‘dry’. Given this variability, the protocol suggests incorporating formal evaluations of the codesign process to help identify limitations, leverage strengths and refine collaboration to ensure maximum benefit [18].

## 3 Discussion

User‐centred development approaches are crucial for creating digital mental health programs that address diverse user needs. However, limited guidance on how to conduct this study meaningfully and efficiently likely hinders user collaboration practices from realising their full potential in enhancing service outcomes [7]. Design Mapping provides a novel conceptual framework for developing digital mental health programs that integrates the creative, user‐centred strengths of Design Thinking, with the rigour and structure of Intervention Mapping. The framework aims to provide an accessible, flexible and practical approach that supports mental health researchers and practitioners to conduct meaningful user codesign, while meeting the demands and constraints of their development environment. Preliminary outcomes from pilot testing of the Active Play program suggest that Design Mapping may support the development of feasible, effective and engaging support programs [45]. While promising, these findings are context‐specific, and further investigation is required to assess the framework's applicability across diverse intervention development settings. Although Design Mapping draws on established evidence‐based methods for intervention development already used in healthcare [4, 24]; the next step is to evaluate its effectiveness relative to alternative development approaches. In the spirit of the iterative design ethos of Design Thinking, this process is expected to lead to further refinement of the framework as new data emerge on its usability and efficacy, and further advancements are made in the field. Ultimately, this study aims to contribute to the establishment of a ‘design science’ – systematic, evidence‐based and formalised design methods that align with the values of empirical scientific inquiry [31].

## Author Contributions

**Kelsie Bufton:** conceptualisation (equal), investigation (equal), methodology (lead), project administration (equal), validation (equal), visualisation (equal), writing – original draft preparation (lead), writing – reviewing and editing (lead). **Maria Bates:** conceptualisation (equal), methodology (support), supervision (equal), investigation (equal), validation (equal), writing – review and editing (support). **Matthew Fuller‐Tyszkiewicz:** conceptualisation (supporting), methodology (supporting), validation (supporting), supervision (supporting), writing – review and editing (supporting). **Jasmin Hamid:** conceptualisation (equal), methodology (supporting), validation (supporting), writing – review and editing (supporting). **Elizabeth Westrupp:** conceptualisation (equal), methodology (supporting), supervision (equal), investigation (equal), validation (equal), visualisation (equal), writing – original draft preparation (supporting), writing – review and editing (supporting).

## Ethics Statement

The research presented in this article was approved by the Deakin University Human Ethics Advisory Group (HEAG‐H 01_2021; HEAG‐H 125_2022).

## Consent

All participants involved in the research pertaining to the current study were provided with a written plain‐language statement outlining the study's purpose and methods and provided informed consent.

## Conflicts of Interest

The authors declare no conflicts of interest.

## Supporting information

Supplementary Material 1.

Supplementary Material 2.

## Data Availability

Data sharing is not applicable to this article as no new data were created or analysed in this study.
