# Supplementary material for: Design Mapping: A Conceptual Framework for Co‐Designing Evidence‐Based Digital Mental Health Programs
Source: Health Expect. 2025 Sep 3;28(5):e70385. doi: 10.1111/hex.70385 (PMC12405966; doi:10.1111/hex.70385)
Supplement: Supplementary file 1 — Supplementary Material 1. [file HEX-28-e70385-s001.pdf]

SUPPLEMENTARY MATERIAL 1. SUPPLEMENTARY TABLES.

Table 1

Comparative Overview of Intervention Mapping and Design Thinking

	Intervention Mapping		Design Thinking
**Pre-Project**	Establish planning group including community members and key stakeholders.	**Pre-Project**	Establish multi-disciplinary team.
**Step 1**	**Needs Assessment:** Establish a detailed understanding of the health problem.<br><br>**Aims:**<br>▪ Identify and describe at risk/target population.<br>▪ Describe the health problem and how it is exhibited in population of interest.<br>▪ Consider behavioural or environmental factors that cause or modify the issue and the determinants of these factors.<br>▪ Determine overall program objectives and intended outcomes.<br><br>**Methods:**<br>• **Primary research:** e.g., interviews, ethnography, surveys, focus groups, delphi technique, planning groups etc.<br>• **Secondary research:** e.g., government archives.	**Phase 1, Step 1**	**Discovery / Inspiration Phase**<br><br>**Aims:**<br>▪ Identify key users and stakeholders.<br>▪ Gain a deep understanding of the problem. Build empathy with the user and discover their explicit and non-explicit needs.<br>▪ Consider a wide range of users and scenarios.<br>▪ Understand the context and history of the design challenge and consider other solutions in the area.<br>▪ Create User Personas to establish the needs of different user groups.<br><br>**Methods:**<br>▪ **Primary research:** Qualitative data collection techniques that enable consumers to be observed behaving naturally, and/or encourage them to open-up and reveal deeper insights. Such techniques include qualitative interviews, user diaries, observations of behaviour.<br>▪ **Secondary research:** Assess effectiveness of recent innovations and solutions in the areas.<br>▪ **Analysis:** Use ‘intuition’ to identify themes in user data.

**Step 2**	**State Expected Program Outcomes and Change Objectives.**<br><br>**Aims**:<br>▪ State the expected change the program aims to bring about in a health-related behaviours and environmental conditions.<br>▪ Divide target behaviours/conditions into performance objectives outlining the specific behaviours or conditions that need to change.<br>▪ Select important and changeable determinants for each performance objective.<br>▪ Set change objectives, or actionable steps, to be taken to influence determinants of performance objectives.	**Phase 1, Step 2**	**Define the Brief**<br><br>**Aims:**<br>• Identify opportunities for design, based on outcomes of a needs assessment.<br>• Create 'Insight Statements': Rephrase each of the themes identified in needs assessment into a statement summarising key insights.<br>• Translate the 'Insight Statements' into 'How Might We' statements that ask how insights might be addressed by the product or service to guide idea generation in the next stage.
**Step 3**	**Select Theory-based Methods and Practical Strategies**<br>**Aims:**<br>• Select or design practical strategies for applying the theoretical methods to the intervention program.<br><br>**Methods:**<br>• Review program ideas with the intended participants and use their perspectives when identifying methods and strategies.	**Phase 2, Step 1**	**Ideation**<br><br>**Aims:**<br>• Conceptualise program solutions.<br>• Create 'Design Principles' by identifying emerging themes in the generated ideas. These principles will guide the various stages of program design.<br><br>**Methods:** A range of strategies are used to foster creativity, collaboration, and idea generation. Comprehensive summaries of Ideation Tools are found in guidebooks offered by design agencies (IDEO, 2015; Victorian State Government, 2020). Example tools include:<br>• Group work incorporating end-users is required to develop complicated services.<br>• Effective and creative brainstorming of solutions enables numerous solutions to be considered and sifted out to arrive at the final design.

			• Visualise Systems and Solutions: Design processes make problems tangible and data visual with sketches and diagrams that convey the relationship between interrelated elements that can easily be altered. Visuals help groups to develop a common understanding of a problem and are promoted for their positive impact on problem-solving.
**Step 4**	**Program: Integrating strategies into organised program**<br><br>**Aims:**<br>• Translate the design into a real, deliverable intervention.<br>• Refine program structure, ensuring logical organisation and feasible delivery.<br>• Draft and pre-test program content to ensure it is effective, feasible, and engaging.<br>• Based on pre-testing, revise program content and proceed to the final production.	**Phase 2, Step 2**	**Prototype, Test, and Refine**<br><br>**Aim:** Create, test, and refine prototypes of program materials.<br><br>**Methods**<br>Prototyping involves several iterative stages of testing and refinement. It might begin with creating and testing simple models, and as each prototype reveals more about what works, iterations become more like a finish product.
**Step 5**	**Adoption and Implementation Plan**<br>Develop strategies and community partnerships to facilitate program adoption, implementation, and maintenance.	**Phase 3**	**Implementation**<br><br>Concepts are developed and refined and a marketing strategy is created. High fidelity prototyping is conducted, followed by user-testing.
**Step 6**	**Evaluation Plan to Measure Program Effectiveness**		

***Note*.** Design Thinking is less prescriptive than the Intervention Mapping approach [1], and no universal develop framework is available. The Design Thinking summary above is a composite of the tools and approaches recommended by several leading design agencies and government department [2-4]. Steps 5 and 6 of Intervention Mapping and Phase 3 of Design Thinking go beyond the scope of the Design Mapping framework and are included in the above table for reference.

Table 2

Examples of Active Play Program Objective, Performance Objectives, Determinants, Theories of How to Influence Change in Determinants, Practical Strategies to Apply Chosen Theories, and Co-design Adjustments.

**Overarching Program Objective: Enhance effective emotion regulation in children aged 2-4 years**				
**Performance Objective**	**Determinant**	**Theory/s**	**Practical Strategy**	**Co-design adjustments**
Positive Parenting Practices.	1. Parent knowledge of effective strategies to support child regulation in different parenting situations.	Active play can be applied in various ways to help children effectively express and regulate their emotions. E.g. (1) Aerobic activity to process activated stress response.	Provide step-by-step guidance on how to facilitate different types of Active Play in different situations: e.g., (1) High energy games when child is dysregulated; (2) Yoga practice to help body to relax.	• Use timer to communicate to the child when to stop playing.<br>• Include cool-down at the end of high-energy games.
	2. Parent to empathise with child emotion.	Psychoeducation on child development to help parent understand child experience.	Brief and relevant information tailored to specific situations.	Validate the difficulties parents encounter in parenting scenarios.
Access and engagement	1. Child willing to participant in Active Play games.	Imaginative play to capture child engagement.	Yoga practice - child pretends to be seed growing into flower.	Imaginative play tailored to child's interests: Lego being built.
	2. Program tailored to needs of individual families.	Personalisation.	Parent encouraged to adapt the game to their child.	Multiple versions of the game provided.
	3. Practical for lives of busy families.	Micro-Intervention.	Short videos addressing specific parenting situations.	Games to be incorporated into existing routines e.g., dressing.
Inclusivity	1. Program inclusive of groups missed by existing support.	Increasing representation of key groups (e.g. fathers, LGBTQIA+ families).	Diverse families represented in the app visuals.	Gender neutral colours and animation style.

Note. Program objectives can be defined as the behaviours/environmental conditions the program want to change; performance objectives are the specific behaviours or conditions that need to change to achieve the program objectives; the changeable determinants are the causal factors that influence achieving the conditions outlined in the performance objectives. Selected theories describe how to influence change in selected determinants, and practical strategies define how to implement these theories.

References

1. Bartholomew Eldredge LK, Markham CM, Ruiter RAC, Fernández ME, Kok G, Parcel GS. *Planning Health Promotion Programs: An Intervention Mapping Approach.* 4th ed. Hoboken, NJ: John Wiley & Sons; 2016. ISBN: 9781119035497.

2. Design Council. *Design for Public Good.* London, UK: Design Council; 2013.

3. IDEO.org. *The Field Guide to Human-Centered Design.* 2015. Accessed August 12, 2025. https://www.designkit.org/resources/1

4. Victorian State Government. *Human-Centred Design Playbook.* Melbourne, Australia: Victorian State Government; 2020.
