# Supplementary material for: Design Mapping: A Conceptual Framework for Co‐Designing Evidence‐Based Digital Mental Health Programs
Source: Health Expect. 2025 Sep 3;28(5):e70385. doi: 10.1111/hex.70385 (PMC12405966; doi:10.1111/hex.70385)
Supplement: Supplementary file 2 — Supplementary Material 2. [file HEX-28-e70385-s002.pdf]

SUPPLEMENTARY MATERIAL 2. DESIGN MAPPING PROTOCOL.

Accompanies: *Co-Designing an Active Play Parenting Program to Support Emotion Regulation in Early Childhood: Presenting User Personas and Program Concepts*

Kelsie Bufton, Maria Bates, Matthew Fuller-Tyszkiewicz, Jamin Hamid, Elizabeth Westrupp

Table of Content

Introduction to the Design Mapping Protocol 2

Pre-project Preparation: Establish a Multi-Disciplinary Team 3

Phase One: Understand 4

- Step One: Needs Assessment 4
- Step Two: Set Program Objectives 9

Phase Two: Co-Develop 12

- Step 1: Establish Program Theory 12
- Step Two: Select and Create Program Strategies and Concepts 14

Phase Three: Prototype (Create, Test, Refine) 18

- Step One: Create Prototypes 19
- Step Two: Test Prototypes 20
- Step Three: Integrate Feedback and Iterate 21

Glossary of Key Terms 22

References 25

Introduction to the Design Mapping Protocol

The following protocol provides a detailed explanation of the three phases of the Design Mapping framework shown in Figure 1, offering a step-by-step methodology to assist those wishing to utilise the approach. The approach seeks to both address limitations in user collaboration practices within the health sector and maintain the rigor and depth of insights required to deliver evidence-based solutions.

First, to support best practice in user collaboration within the healthcare sector, Design Mapping integrates methods to involve end-users in the initial stages of content development to ensure solutions align with their needs [1]; provides guidance for facilitating creative ideation [2]; includes tools for meaningfully identifying and addressing variation in user needs; and offers practical tools to support the continued utilisation of codesign outcomes throughout all stages of program development.

Second, to ensure methodological rigour and depth of insights-[3], the Design Mapping framework considers ways to balance user driven insights with clinical expertise and empirical evidence, ensuring scientific rigor is not compromised in favour of usability [4]; presents a robust mixed-methods approach to participant recruitment, data collection, and analysis, grounding program development in reliable research outputs; and incorporates methods for assessing the acceptability, feasibility, and validity of codesign practices, enabling meaningful evaluation and refinement of collaboration efforts for maximum benefit [1, 3]. The framework also offers flexibility across the design process, proposing a gold standard for maximising engagement and efficacy, while offering options to adapt this standard to accommodate common constraints, such as funding and grant parameters, and limited resources. This flexibility acknowledges the realities of intervention development in the health sector and aims to elevate the overall quantity and quality of codesign in the field by guiding teams to make pragmatic concessions in ways that minimise impact on the quality of outcomes [3, 5].

Figure 1

Design Mapping: A Framework for Developing Digital Mental Health Programs, Drawing Upon Design Thinking and Intervention Mapping

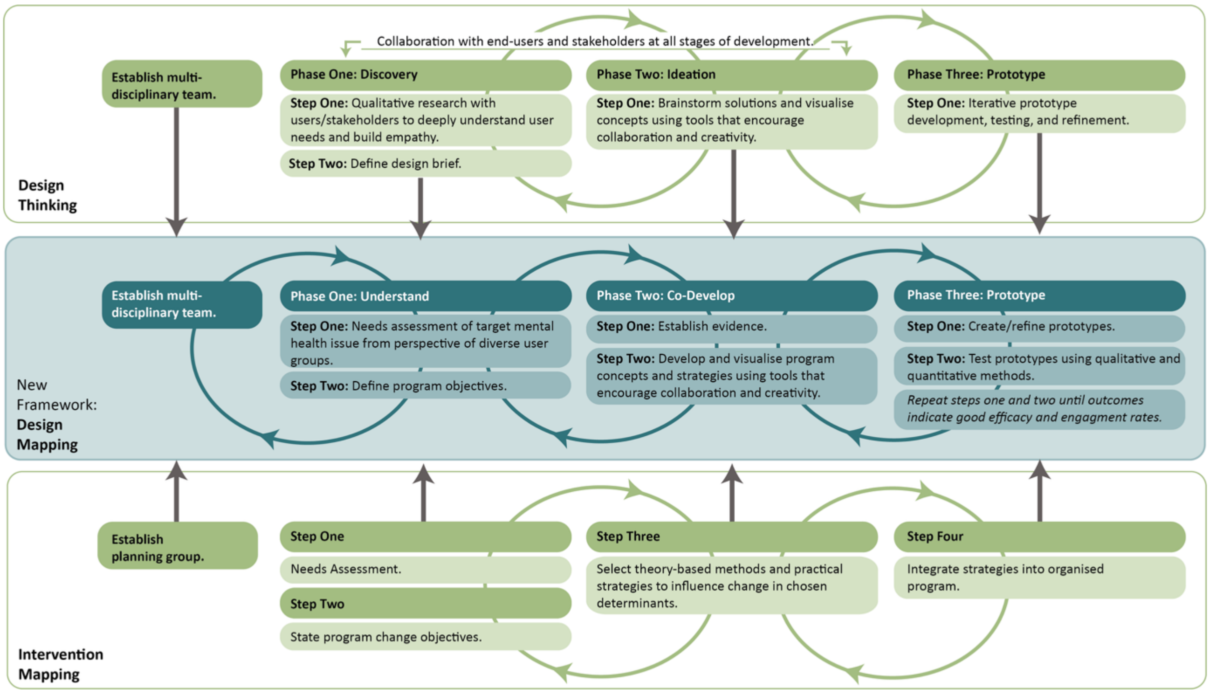

Pre-project Preparation: Establish a Multi-Disciplinary Team

As per Design Thinking and Intervention Mapping [6, 7], Design Mapping recommends establishing a multi-disciplinary program development team prior to project commencement, which may evolve as development needs change. This group may consist of a core program development team, with the capacity to play a central role in all areas of development; and advisory members who are brought in to offer expert perspective as needed. Team composition should ensure adequate representation of the core project areas, so that collective knowledge can effectively address the project's complexity, including its technical, design, business, and human dimensions [8]. Design Reference Groups can also be established at the beginning of the project comprising of diverse user types and/or stakeholders. This group can be consulted throughout development on a range of areas, including reviewing recruitment strategy, research insights, and marketing materials, to anchor processes in user articulated needs and preferences [9]. Team members should be oriented to the codesign philosophy and process to establish an alignment of values and a shared commitment to

developing a program that reflects user needs, rather than top-down expertise [10]. This process may entail reflexive practice to address researchers' subjectivity in interpreting and responding to user data [11].

Phase One: Understand

Table 1
Summary of Phase One

Step	Aim	Key Methods/Tools
1. Needs Assessment	• Define and understand the target mental health issue.<br>• Define and understand the target user.<br>• Explore user perceptions of digital technology.<br>• Establish status of program solutions.	• Secondary data: Literature review.<br>• Primary Data: Qualitative research e.g., user Interviews, workshops, video diaries, ethnography.<br>• Robust analysis techniques e.g., Thematic analysis.<br>• Outputs: User personas.
2. Set Program Objectives	Establish overall program objectives, specific performance objectives, and changeable determinants.	• Team collaboration/brainstorming sessions.

Step One: Needs Assessment

As per Intervention Mapping and Design Thinking [6, 7], the first phase of Design Mapping involves defining and assessing the target mental health problem. When creating digital mental health programs, developers may have a predetermined target mental health issue, be it a diagnosis or a problem behaviour, as well as preconceived ideas about how a digital program might address these target issues [12]. Regardless of prior knowledge, a needs assessment should be conducted to (1) gain a comprehensive understanding of the causes and impact of the target mental health issues affecting the target population; (2) identify existing and required supports; and (3) recognise potential barriers to program feasibility, acceptability, and engagement from the perspective of diverse target users. This approach ensures that early program ideas are selected, refined, and challenged, to align with a deep understanding of the needs and experiences of the program's

intended users [6, 13]. While the specific approach will vary from project to project, a needs assessment may address the following aims [6, 11, 14]:

Aims

1. **Understand the problem.** Drawing on Intervention Mapping, the assessment should seek to describe (1) the target mental health issue; (2) the impact the mental health issue has on quality of life for those affected; (3) the biological, psychological, and social factors that precipitate and perpetuate the mental health problem, including how the issue relates to the environment or system in which it exists; and (4) the determinants of these factors [6].
2. **Define and understand the target user.** The assessment may also involve defining and understanding the needs and experiences of the target users, identifying sub-groups within the broader user population, including those often missed by existing mental health supports [15]. Once user groups have been identified, it can be beneficial to seek a first-hand account from each group regarding the impact of the target mental health issue, and their unmet needs in relation to mental health supports. We note a recent shift in the conceptualisation and terminology used to describe people or groups who experience health inequalities, and/or a disproportionate burden of poor health. Terms used in Intervention Mapping and Design Thinking such as 'at risk,' 'vulnerable,' or 'extreme' groups can imply an intrinsic deficit that supersedes other explanations for the circumstances under consideration. These deficit terms can be disempowering and disrespectful, given that societal and systemic power structures typically determine access to resources and support services. While it can be beneficial to identify groups of people disproportionately impacted by health issues, deeper consideration should be paid to the characteristics or circumstances that place them at increased risk of harm, which may span different demographic groups [16]. To ensure the sensitive identification and engagement of users, as well as a nuanced understanding of their needs and experiences, the development team may require additional consultation and training, especially in areas such as cultural competence, mental health awareness, and trauma informed practice [6].

Identifying both commonalities and variations in user experiences and characteristics can help create solutions that effectively address diversity within shared user needs. Commonality can be represented as a set of themes derived from both primary and secondary data sources [7]. Diversity within these themes can then be represented by a set of user personas. User personas are symbolic representations of a 'typical' user group, illustrating their needs, behavioural patterns, experiences, and reactions. Personas help clarify the target end-users, build empathy with them, and ensure that solutions are designed to cater to diversity in their needs [8]. In Design Mapping, we suggest conceptualising user personas based on shared values, beliefs, experiences, and motivations. While demographic factors (e.g., age) or family composition (e.g., single parent) may be included if pertinent, a deeper approach helps to target underlying factors that contribute to engagement and effectiveness for a specific user group, and helps avoid perpetuating unhelpful stereotypes [17]. In line with Design Thinking, it is recommended to create at least one persona representing individuals most likely to be overlooked by a future program [14]. Developers can then prioritise engaging these overlooked personas in subsequent development work. To enhance productivity, focus should be placed on engaging users who are receptive to digital support, provided their needs can be adequately addressed.

In many instances, the target user will comprise of people experiencing the target mental health issue(s). In other cases, recipients may include those who support people experiencing, or at risk of experiencing, mental health difficulties, including mental health practitioners, local government and community organisations and workers, families and carers, teachers, early childhood educators, etc. In such cases, the needs and experiences of both the program users and those experiencing mental health difficulties may be considered.

3. **Explore users' perceptions of digital technology as a medium for mental health support.** The needs assessment may also seek to identify ways a digital mental health program can be engaging, acceptable, and feasible for diverse users. It may be beneficial to understand the

performance of existing digital mental health programs in the field, including how effectively they engaged consumers and have influenced change on target mental health outcomes. This review should be based on up-to-date recommendations on engagement metrics and benchmarks e.g. see Bijkerk, Oenema [18] for more information.

4. **Establish the status of program solutions.** In Intervention Mapping, a needs assessment precedes the selection of program solutions [6]. In contrast, Design Mapping allows for a more flexible approach. When solutions are pre-selected—due to prior work or external constraints—developers may instead focus on evaluating their evidence base alongside users' perceptions and experiences. This approach enables the early identification and resolution of potential barriers that may limit program efficacy. It is recommended that steps are taken to reduce bias towards preferred solutions, such as using neutral, open-ended questions during user research, and limiting the disclosure of information about known program elements to participants. Collaborative analysis of research data can also enhance bias detection [19].

Methods

A range of methods may be relevant, with the specific approach tailored to the needs of the project and how much pre-existing data there is in the field.

1. **Secondary data.** A literature review is valuable for identifying published prevalence rates; characteristics of individuals at risk; determinants that influence the onset and maintenance of mental health issues; and reviewing evidence supporting pre-selected program solutions. This data can help support diverse and representative participant recruitment and inform which topics are explored in qualitative user research.
2. **Primary data.**

- Qualitative research can help the development team build empathy with the target end-user, and understand their needs, beliefs, and experiences within the context of the target mental health issue.

- Recruiting a diverse sample of end-users and stakeholders will provide a nuanced understanding of the impact of the target mental health issue [11]. Ideally, the sample of end-users should be representative of those at population level, including groups commonly missed by existing programs. Sufficient time should be allocated to ensure adequate representation of people living with mental health issues, which can be time-consuming when recruiting from communities who face engagement barriers. To support efficient recruitment of diverse user groups, it may be beneficial to establish partnerships with relevant community and clinical organisations early into the development process, and to recruit participants, and conduct qualitative research virtually [20]. Snowball sampling can also assist in reaching specific demographics through targeted referrals from existing participants, creating a chain-like expansion of the sample. However, this method should be used sparingly to minimise bias [21]. Principles and tools for sensitive research engagement should also be considered e.g., see Moll, Wyndham-West [11].
- Design Thinking handbooks recommend a variety of qualitative data collection tools to uncover user needs, however, do not typically suggest engaging in formal, standardised qualitative data analysis techniques [14]. Design Mapping encourages the use of immersive qualitative tools, such as interviews, ethnography, video diaries, workshops, and focus groups, and recommends that research data is recorded and analysed using robust qualitative analysis techniques that enable common themes and diversity in user experience to be accurately understood and represented. For example, thematic template analysis is a flexible and iterative method used to identify themes within qualitative data. While the approach can be time consuming, it is relatively simple to apply for those new to qualitative research [22]. In projects with limited resources, qualitative research may benefit from addressing specific research questions relating to addressing unmet user needs.

A Note on Pre-defined Project Parameters

While Design Mapping allows for certain project parameters to be pre-defined by developers, it also aims to arrive at solutions that are desirable, feasible, and viable for the target end-user. Therefore, the needs assessment should be open to different solutions emerging as the understanding of problem and user needs deepens, particularly when pre-identified solutions conflict with user articulated preferences [13]. Developers may resolve such tensions by re-evaluating the evidence and rationale behind pre-selected components, and if deciding to proceed, taking steps later in development to adjust how these components are applied to accommodate user preferences.

Step Two: Set Program Objectives

In line with both Intervention Mapping and Design Thinking, Design Mapping suggests defining development objectives based on the outcomes of the needs assessment [6, 7]. Ideally all program objectives will be informed by the outcomes of the assessment, however Design Mapping recognises that in some projects certain objectives may be predefined based on prior consideration of the research literature e.g., project funding may be tied to the use of a specific therapy to address a specific mental health issue. In these instances, the development team are encouraged to set additional objectives informed by the outcomes of the needs assessment, which may include addressing the limitations of preselected strategies identified during a literature review. In line with Intervention Mapping, it is recommended that three tiers of program objectives are set [6]:

Aims

1. **Define overall program objectives/opportunities for design.** Overall, what behaviours or environmental conditions does the program aim to change?
2. **Define performance objective.** What are the specific behaviours or conditions that need to change to achieve the overall program objectives? These objectives may be informed by both existing empirical literature and qualitative research conducted with end-users. When setting objectives, developers may prioritise effectively addressing mental health outcomes. However,

as engagement is a key mechanism in predicting the treatment benefits obtained [23], developers may also wish to address outcomes that pose access, engagement, and feasibility challenges. These objectives can address the general barriers to receiving mental health treatment, and those specific to digital platforms, such as data privacy, and ethics of data collection [24]. In line with Design Thinking, it is recommended that objectives also aim to meet the needs of the different personas, focusing on those experiencing most engagement barriers [14].

3. **Define changeable determinants.** Developers are encouraged to identify causal factors that influence achieving the health-promoting and program engagement behaviours, and environmental conditions outlined in the performance objectives. Determinants can be identified by cross-referencing outcomes of user research with relevant research literature. Changeable determinants might include personal determinants, such as an individual's skills, knowledge, beliefs, values, self-efficacy, and expectations, and external determinants, such as social norms and supports, access to resources, policies, and organisational climates [6].

Methods

Creating a list of determinants may occur iteratively over a series of discussions and collaboration sessions among the project development team. These sessions may begin by considering many ways of looking at the problem, before resolving into a single brief [14]. When selecting the final list of target determinants, group members are encouraged to consider both their relevancy and change potential [6]. Collaboration sessions, including those involving end users, can benefit from visual brainstorming exercises.

Tips for Effective Brainstorming. Brainstorming sessions should aim to gather multiple perspectives and solutions to a defined problem [8]. Subsequently, these ideas can be refined by clustering them based on themes and patterns, discarding unsuitable ones, and translating them into more complex solutions [7]. To encourage creativity and the unrestrained generation of ideas, facilitators are recommended to pay attention to conditions that promote effective brainstorming. In

sessions with inherent power imbalances, such as when participants include both end-users and industry practitioners, or have varying seniority levels within teams, participants should be empowered to contribute as equals. This might involve encouraging managers to take a back seat or utilising small group work. Design agencies, such as IDEO, provide comprehensive guidelines for effective brainstorming, some of which are summarised below:

- Set a clear session objective: "How might we use (theory x) to address (factor y)?"
- Hold your criticisms: To help everyone feel safe sharing thoughts, don't judge, or argue about ideas at first. Ideas can be critiqued when you later distil them.
- Think outside the box: Encourage imaginative ideas, as this can lead to creative solutions and encourage everyone to think without limits.
- Generate lots of ideas: Generate the highest number of ideas you can think of.
- Draw your thoughts: Write or sketch ideas where everyone can see them, such as on a digital screen, butcher paper, or using sticky notes.
- Warm up: Start with a warm-up exercise if the group is new, doesn't brainstorm often, or seems distracted [7].

Phase Two: Co-Develop

Table 2
Summary of Phase Two

Step	Aim	Key Methods/Tools
1. Establish Evidence.	Identify theoretical methods that influence key change mechanisms.	• Literature review.<br>• Stakeholder Consultation.<br>• Output: Design Principles / Logic Models.
2. Develop Concepts and Strateiges.	• Develop practical strategies for applying theoretical methods.<br>• Organise strategies into program concepts.	• “How might we” statements.<br>• Ideation Workshops.<br>• Codesign Workshops.<br>• Visualisation tools e.g., Journey Mapping, Storyboards.

Step 1: Establish Program Theory

Aims

In line with Intervention Mapping, the next stage of Design Mapping involves identifying theoretical methods that can influence change in the selected determinants of mental health promoting behaviours and environmental conditions (e.g., knowledge, skills, access to resources).

Methods

1. **Review literature to identify relevant theoretical methods.** Intervention Mapping offers extensive examples of how behaviour change theories can be applied to create health programs [6], and others have considered behaviour change stategies specifically from the perspective of developing digital health initiatives [25]. In Design Mapping, developers are encouraged to consider whether selected behaviour change theories or therapeutic models are engaging and suitable for digital content. For example, there is evidence that goal setting and fostering social support positively influences engagement with digital mental health programs [26]. Additionally, developers are encouraged to identify theories on enhancing engagement through the delivery of digital content, such as personalization, professional guidance and support, and design. As

such theories have been the subject of several reviews, they will not be discussed in detail here. For further information, see [26-29].

2. **Stakeholders Consultation.** Alongside a literature review, end-users, relevant stakeholders, and industry experts may also be consulted to identify and determine the suitability of selected theories, especially if literature is lacking in the area. The suitability of different theories may be considered in light of how well they meet the needs of the target user personas. If some theoretical methods are predetermined, a literature review should consider factors that may enhance the efficacy and engagement of the chosen theory, based on the outcomes of needs assessment. For example, the primary theoretical model may be predefined as 'Cognitive Behavioural Therapy (CBT)' to treat depression, however, an additional theory of 'providing psychoeducation about depression' might be identified to normalise help seeking for a persona experiencing self-stigma.
3. **Create design principles / logic models.** Design Thinking suggests that as ideas evolve, unifying elements emerge to guide the design process. These can be translated into a set of "design principles" that describe the program's most important components. These principles help give integrity and form to the program, ensure that important elements and concepts are not lost as the program evolves, and inspire creative decisions about how program content is delivered [7]. In Design Mapping, we suggest that design principles can be created to capture the projects key theoretical concepts that may also align with themes identified in the needs assessment. An alternative/supplementary approach is to create a logic model or diagram (also referred to as 'program logic') to communicate the different theoretical concepts of how the program is proposed to work, including the theoretical mechanisms via which a program influences proposed outcomes [13]. Design principles and logic models should be co-developed by the development team in collaboration sessions that draw upon the brainstorming guidelines outlined above and can be updated and evolved as development progresses [7, 30].

Step Two: Co-Create

Aims

1. **Select and design practical strategies for applying theoretical methods.** According to Intervention Mapping, a practical strategy is a specific technique employed to implement theoretical methods that is suitable for the program population and the context in which the intervention will be conducted [6]. The Design Mapping framework suggests that the exact approach of how practical solutions are selected and designed can be tailored to the needs and scope of the specific project using Design Thinking tools that encourage creativity and collaboration [7, 30]. In some projects, existing strategies will be incorporated and in others new strategies will need to be designed. As digital technology is continuously evolving, developers are encouraged to review the literature and consult with software engineers to identify innovative and appropriate strategies for delivering digital programs in accordance with selected theories [31]. For example, machine learning can be used to personalise inputs and outputs based upon a person's situation, characteristics, and desired outcomes [29]. Additionally, online discussion forums and gamification elements, such as leader boards, can provide social and professional support [26].
2. **Organise selected strategies into program concept/s.** Both Design Thinking and Intervention Mapping suggest that once the program strategies have been selected, they should be refined and translated into a program concept that is logically ordered, feasible to deliver, and addresses the program objectives [6, 7]. We suggest that in complex projects, an overall project concept may need to be created, with several sub-concepts siting below. It is recommended that the process of creating a program concept draws upon empirical research literature, knowledge of related digital programs, ideas generated during collaboration sessions, and design principles/logic models.

Methods

In Design Mapping, we suggest that the approach for selecting and creating practical strategies and program concepts can be tailored to the specific project, and may benefit from incorporating the following Design Thinking concepts [8]:

1. **“How might we” statements.** “How might we” statements are leading questions that can help focus the team on an outcome. Creating a set of statements reflecting the practical solutions that need identifying to address chosen determinants can help focus collaboration sessions, and act as a gauge for when sufficient solutions have been identified. A set of “how might we” statements can be created by considering selected theories alongside changeable determinants e.g., “How might we provide psychoeducation [theory] to reduce stigma [determinant]?” [7, 30].
2. **Collaborative, multi-disciplinary approach to strategy selection and concept design.** Theoretical methods can be applied via a range of practical solutions that, in turn, can be adapted in endless ways [6]. To determine the best approach, it is recommended that practical solutions are selected, designed, and refined into concepts collaboratively between members of the program development team, considering the constraints of the specific project and the needs of different personas. This process is likely to occur iteratively over several collaboration sessions that aim to gather multiple perspectives on a defined problem, drawing on the guidelines for effective brainstorming outlined above [8]. Initially, sessions may focus on generating as many ideas as possible. Later, program strategies can be refined by clustering ideas based on themes and patterns, discarding unsuitable ideas, and crafting multi-dimensional concepts [7]. To ensure the technical, research, and human dimensions of a problem are all represented, ideally members of the development team from various disciplines should participate in the selection and development of strategies.
3. **User input.** Design Thinking emphasises codesigning program strategies and concepts in collaboration with end users and relevant stakeholders [14]. While Design Mapping

acknowledges the importance of user input during content development, it also recognises that in evidence-based mental health programs the theoretical methods underpinning the program, and some of their associated practical components, are grounded in empirical evidence and practitioner expertise, and therefore inflexible to user input. However, where possible, developers should seek input from end-users and other relevant stakeholders during the development of the program strategies to ensure they are accessible, engaging, and feasible. Users can offer insight into how or when the content or delivery of a digital program might fail to meet their needs or lose their engagement and be involved in designing solutions to identified issues. Users can be presented with specific “how might we” statements asking them to address a specific element of the design (e.g., “How might we make the design aspects of a digital CBT program engaging for a depressed teen?). User ideas are valuable for providing practical perspectives that may have been overlooked by the development team and can be filtered based on their alignment with the empirical research literature. User input can also include early input on how the digital program is branded and how the content is delivered, considering components such as language, tone, animation style, and colour schemes [7, 30].

As user experience of the codesign process is likely to vary from one project to another, it is recommended that a brief assessment of the acceptability, feasibility, and validity of codesign processes are conducted so that future collaboration efforts in the project can be fine-tuned [1]. This assessment might be conducted through brief feedback exercises or questionnaires distributed at the end of qualitative methods. While the availability of robust quantitative evaluation tools is still evolving, an example feedback survey can be found in the Experience-Based Codesign Toolkit [32].

4. **Visualise solutions.** Design Thinking suggests that when creating a program concept, it can be helpful to move from abstract thinking to visualising how practical solutions come together [8]. An overview of various useful visualisation tools is available in handbooks offered by leading design agencies, e.g., see [7, 14, 30]. One commonly used and accessible practice is the creation

of ‘journey maps’ that visualise a user’s interaction with a product or service from start to finish. Journey maps can visualise how users interact with an existing product or service or depict a developer’s vision for the user experience they want to create. Maps can visualise the user experience when engaging with a program from start to finish, during a single instance of engagement, or when implementing or interacting with a specific strategy or resource. The journeys of different personas can also be considered, enabling designers to consider ways to meet the needs of different groups early in design [7]. Journey maps can also serve as early prototypes to test program concepts and strategies with end users and stakeholders. In codesign sessions, developers might ask participants to identify "pain points" in the user journey at both macro and micro levels. A ‘pain point’ refers to an issue, problem, or difficult moment in a user’s experience that may evoke negative emotions or perceptions (e.g., they perceive judgement and feel shame) and be detrimental to program efficacy and engagement [30]. Participants can contribute to generating solutions to identified pain points by brainstorming alternative solutions, creating simple prototypes of alternatives, or role-playing different scenarios. Once a concept has been created, it may be helpful to visualise it as an advanced iteration of the logic model created in phase two, step two, that depicts the various theories and strategies of the program.

Phase Three: Prototype (Create, Test, Refine)

Table 3
Summary of Phase Three

Step	Aim	Key Methods/Tools
1. Create Prototypes	Develop early versions of program components for testing.	• Low fidelity prototypes.
2. Test Prototypes	Evaluate feasibility, usability, and engagement of program components and concepts.	• Mixed method testing (qualitative and quantitative) e.g., pilot testing via randomised control trial, interviews, workshops.
3. Refine Prototypes	Revise and refine program components, based on testing outcomes.	• Collaborative prototype refinement, drawing upon logic models, design principles, user personas. • Additional testing as required.

Prototyping is a central process in Design Thinking, enabling abstract program elements to be translated into tangible forms, which can be tested with end-users and key stakeholders, and refined iteratively based on feedback. The program theory and logic models are important at this point, and developers may test whether some of their proposed behaviour change mechanisms positively impact intermediate outcomes, if statistical power allows [13, 14].

In Design Mapping, we recommend that the prototyping approach is tailored to the needs and resources of the specific project. Ideally, several rounds of prototyping will be conducted, where the below three iterative steps are repeated until an optimal solution is achieved [7]. The process can begin with the creation of inexpensive, low-fidelity prototypes that can be tested using simple cost-effective research methods. As each prototype reveals more about what works, iterations become more like a finished product that can undergo usability testing using more rigorous and robust quantitative research methods, potentially leading to a pilot test of a digital program using a randomized control design. This testing strategy helps establish empirical evidence of the program's

efficacy and engagement, and is an extension of the Design Thinking methodology, which generally excludes the testing of prototypes with quantitative methods [6]. While prototyping can appear labour intensive, the iterative process of testing and refining ideas keeps end-users at the centre of the design process. This approach enhances efficiency and reduces costs by enabling unintended consequences, or risk of failure, to be identified and ‘designed out,' resulting in a final program that is more likely to be adopted by end-users [7, 8, 14].

When digital mental health programs introduce novel concepts or strategies, developers may choose to begin the prototyping process by gathering user feedback on low-fidelity prototypes, such as scripts or diagrams, before later progressing to test high-fidelity digital resources integrated into a digital platform. In contrast, programs following established methods and strategies may opt to start with more evolved prototypes [7, 8, 14].

Step One: Create Prototypes

Aim

Create prototypes of key program components that enable their efficacy, engagement, and feasibility to be tested.

Methods

The content of digital health programs often includes multiple testable components. To determine what to prototype, the development team may identify what still needs to be understood about the most pertinent program elements. Developers may choose to prototype the whole program, or individual touchpoints, such as forms, web pages, or videos. Prototypes can include drawings or diagrams of program concepts; simplified versions of digital resources; role-playing how a user might interact with the program or implement a specific strategy; storyboarding a particular element of the program; or live testing of the digital platform [7].

Step Two: Test Prototypes

Aim

Acquire feedback on prototypes from end users, stakeholders, and professionals to determine how the efficacy, engagement, and feasibility of the program can be improved.

Methods

It is recommended that prototypes are tested iteratively using a mixed methods approach with diverse users and stakeholders, including groups commonly missed by existing programs. Prototype testing can investigate the usability of program content and seek feedback on content delivery, assessing design elements, language, and tone of voice etc [7].

- Qualitative methods, such as interviews, ethnography, and workshops, are particularly valuable during early development stages. These methods are less likely to be biased by developers' interests, encourage open participant feedback on matters most pertinent to their needs, and enable collaborative problem solving to address identified issues [2]. For example, a codesign workshop run with end-users might include "rapid prototyping" activities, where users generate updated prototypes through storyboarding and roleplays [7].
- Quantitative methods during the evaluation of later-stage prototypes enable researchers to acquire feedback on specific elements of interest, and help establish empirical evidence regarding program effectiveness, acceptability, engagement, and feasibility at population level [33, 34]. It is therefore recommended that prototype testing culminate in a quantitative pilot study of the digital program using a randomised control design. For such testing to be effective and reliable, sample sizes should be sufficiently large [35].

Step Three: Integrate Feedback and Iterate

Aims

Integrate prototype testing outcomes into a further iteration of program components.

Methods

It is recommended that key findings from prototype testing are summarised and presented back to the development team, alongside user personas, design principles, and logic models, which may require updating based on the outcomes of prototype testing. Subsequently, the development team may collaboratively brainstorm to integrate findings into updated prototypes. If further questions remain, further prototype testing can be conducted until outcomes indicate the program is effective and likely to be adopted by end-users [7].

Glossary of Key Terms

Brainstorming: A structured group process used to generate a large number of creative ideas, guided by principles such as deferring judgment, encouraging quantity, and building on others' ideas. May utilise visual prompts like "how might we" statements [7].

Changeable Determinants: Personal or environmental factors (e.g., beliefs, skills, social norms) identified as both influential and modifiable through program strategies [6].

Codesign: Codesign is a collaborative method of 'collective creativity' or 'partnership' with target end users, who participate in all stages of product/service development to ensure that it aligns with their needs and preferences [1].

Codesign Workshop: Codesign workshops are used to facilitate user collaboration, and involve members of the design team, stakeholders, and users working together to generate ideas and solutions to a shared problem [30]

Design Principles: Core ideas derived from theoretical models and user research that guide program design decisions, ensuring consistency and alignment with user needs and values [7].

Design Thinking: An iterative, non-linear, approach to product and service development that prioritises usability, collaboration, innovation, and prototyping [5, 36].

Design Reference Group: A group of stakeholders, often including end-users, convened to provide input and feedback throughout the program development process [9].

Determinants: Factors that influence whether a target behaviour or environmental condition occurs. These may include skills, attitudes, knowledge, social norms, or external barriers [6].

How Might We Statements: Open-ended prompts that frame design challenges as opportunities for innovation (e.g., "How might we reduce stigma using psychoeducation?"). Can be used to structure brainstorming and ideation [6].

Ideation Workshop: Ideation workshops bring members of the program development team and stakeholders together to creatively work with research insights, and generate a diverse range of potential solutions to a design issue [30].

Intervention Mapping: A systematic step-by-step protocol for developing, implementing, and evaluating evidence-based health promotion programs [6].

Logic Model (Program Logic): A visual or narrative representation of how a program is intended to work, mapping inputs, strategies, theoretical mechanisms, and expected outcomes [13].

Multidisciplinary Program Development Team: A team composed of individuals from diverse professional backgrounds (e.g., clinical, research, design, engineering) who contribute varied expertise to program development [6, 7].

Needs assessment: A systematic process used to examine a health problem within a specific population by identifying its known causes, associated risk behaviours, and environmental factors, serving as the foundation for developing effective health promotion interventions [6].

Pain point: An issue, problem, or difficult moment in a user's experience that may evoke negative emotions or perceptions (e.g., they perceive judgement and feel shame), and can therefore be detrimental to program efficacy and engagement [7, 30].

Performance Objectives: Specific, measurable goals that define what users or environments need to change to meet the overall aims of a program [6].

Predefined Solutions: Program components or strategies selected before user input due to prior research, funding conditions, or institutional priorities. These components may still be refined through codesign.

Program Concept: An overarching design vision that integrates strategies, content, and delivery into a coherent structure aligned with program objectives [7, 30].

Prototype: A tangible, testable version of a program element (e.g., wireframe, mock-up, script) used to evaluate efficacy, feasibility, and engagement, and improve design [7, 14, 30].

Thematic Template Analysis: A qualitative method used to identify themes in qualitative data. Offers a flexible, structured approach to coding and analysing user data [37].

User journey map: Used to visualise a user's interaction with a product or service from start to finish. Journey maps can visualise how users interact with an existing product or service or depict

a developer’s vision for the user experience they want to create. They can visualise the user experience when engaging with a program from start to finish, during a single instance of engagement, or when implementing or interacting with a specific strategy or resource [7]

User personas: Fictional archetypes that illustrate the characteristics of 'typical' users within end-user groups [7, 14].

References

1. Bevan-Jones R, Stallard P, Agha SS, Rice S, Werner-Seidler A, Stasiak K, et al. Practitioner review: Co-design of digital mental health technologies with children and young people. Journal of Child Psychology and Psychiatry. 2020;61(8):928-940. doi:10.1111/jcpp.13258.
2. Bird M, Ouellette C, Whitmore C, Li L, Nair K, McGillion M, et al. Preparing for patient partnership: A scoping review of patient partner engagement and evaluation in research. Health Expectations. 2020;23(3):523-539. doi:10.1111/hex.13040.
3. Bazzano AN, Martin J, Hicks E, Faughnan M, Murphy L. Human-centred design in global health: A scoping review of applications and contexts. PLoS One. 2017;12(11):e0186744. doi:10.1371/journal.pone.0186744.
4. Slattery P, Saeri AK, Bragge P. Research codesign in health: A rapid overview of reviews. Health Research Policy and Systems. 2020;18(1):17. doi:10.1186/s12961-020-0528-9.
5. Oliveira M, Zancul E, Fleury AL. Design thinking as an approach for innovation in healthcare: Systematic review and research avenues. BMJ Innovations. 2021;7(2):491-498. doi:10.1136/bmjinnov-2020-000428.
6. Bartholomew Eldredge LK, Markham CM, Ruiter RAC, Fernández ME, Kok G, Parcel GS. Planning health promotion programs: An intervention mapping approach. 4th ed. Hoboken, NJ: John Wiley & Sons; 2016. ISBN: 9781119035472.
7. IDEO. Field guide to human-centered design. 2015. Available from: https://www.designkit.org/resources/1. Accessed August 12, 2025.
8. Micheli P, Wilner SJS, Bhatti SH, Mura M, Beverland MB. Doing design thinking: Conceptual review, synthesis, and research agenda. Journal of Product Innovation Management. 2019;36(2):124-148. doi:10.1111/jpim.12468.
9. Lammers J, Happell B. Research involving mental health consumers and carers: A reference group approach. International Journal of Mental Health Nursing. 2004;13(4):262-266. doi:10.1111/j.1447-0349.2004.00339.x.

10. Baines R, Bradwell H, Edwards K, Stevens S, Prime S, Tredinnick-Rowe J, et al. Meaningful patient and public involvement in digital health innovation, implementation and evaluation: A systematic review. Health Expectations. 2022;25(4):1232-1245. doi:10.1111/hex.13506.
11. Moll S, Wyndham-West M, Mulvale G, Park S, Buettgen A, Phoenix M, et al. Are you really doing 'codesign'? Critical reflections when working with vulnerable populations. BMJ Open. 2020;10(11):e038339. doi:10.1136/bmjopen-2020-038339.
12. de Beurs D, van Bruinessen I, Noordman J, Friele R, van Dulmen S. Active involvement of end users when developing web-based mental health interventions. Frontiers in Psychiatry. 2017;8:72. doi:10.3389/fpsyt.2017.00072.
13. O'Cathain A, Croot L, Duncan E, Rousseau N, Sworn K, Turner KM, et al. Guidance on how to develop complex interventions to improve health and healthcare. BMJ Open. 2019;9(8):e029954. doi:10.1136/bmjopen-2019-029954.
14. Design Council. Design for public good. 2013. Available from: https://www.designcouncil.org.uk/resources/report/design-public-good. Accessed August 12, 2025.
15. Khalid HM. Embracing diversity in user needs for affective design. Applied Ergonomics. 2006;37(4):409-418. doi:10.1016/j.apergo.2005.08.009.
16. Munari SC, Wilson AN, Blow NJ, Homer CSE, Ward JE. Rethinking the use of 'vulnerable'. Australian and New Zealand Journal of Public Health. 2021;45(3):197-199. doi:10.1111/1753-6405.13098.
17. Laubheimer P. Personas vs. archetypes. Nielsen Norman Group. 2022. Available from: https://www.nngroup.com/articles/personas-archetypes/. Accessed August 12, 2025.
18. Bijkerk LE, Oenema A, Geschwind N, Spigt M. Measuring engagement with mental health and behavior change interventions: An integrative review of methods and instruments. International Journal of Behavioral Medicine. 2023;30(2):155-166. doi:10.1007/s12529-022-10127-8.

19. Morse JM, Barrett M, Mayan M, Olson K, Spiers J. Verification strategies for establishing reliability and validity in qualitative research. International Journal of Qualitative Methods. 2002;1(2):13-22. doi:10.1177/160940690200100202.
20. Han J, Torok M, Gale N, Wong QJ, Werner-Seidler A, Hetrick SE, et al. Use of web conferencing technology for conducting online focus groups among young people with lived experience of suicidal thoughts: Mixed methods research. JMIR Mental Health. 2019;6(10):e14191. doi:10.2196/14191.
21. Sadler GR, Lee HC, Lim RSH, Fullerton J. Recruitment of hard-to-reach population subgroups via adaptations of the snowball sampling strategy. Nursing & Health Sciences. 2010;12(3):369-374. doi:10.1111/j.1442-2018.2010.00541.x.
22. Clarke V, Braun V. Successful qualitative research: A practical guide for beginners. London: SAGE Publications; 2013.
23. Gan DZQ, McGillivray L, Han J, Christensen H, Torok M. Effect of engagement with digital interventions on mental health outcomes: A systematic review and meta-analysis. Frontiers in Digital Health. 2021;3:764079. doi:10.3389/fdgth.2021.764079.
24. Barnett S, Huckvale K, Christensen H, Venkatesh S, Mouzakis K, Vasa R. Intelligent Sensing to Inform and Learn (InSTIL): A scalable and governance-aware platform for universal, smartphone-based digital phenotyping for research and clinical applications. Journal of Medical Internet Research. 2019;21(11):e16399. doi:10.2196/16399.
25. Mohr DC, Schueller SM, Montague E, Burns MN, Rashidi P. The behavioral intervention technology model: An integrated conceptual and technological framework for eHealth and mHealth interventions. Journal of Medical Internet Research. 2014;16(6):e146. doi:10.2196/jmir.3077.
26. Perski O, Blandford A, West R, Michie S. Conceptualising engagement with digital behaviour change interventions: A systematic review using principles from critical interpretive

synthesis. Translational Behavioral Medicine. 2017;7(2):254-267. doi:10.1007/s13142-016-0453-1.

27. Yardley L, Spring BJ, Riper H, Morrison LG, Crane DH, Curtis K, et al. Understanding and promoting effective engagement with digital behavior change interventions. American Journal of Preventive Medicine. 2016;51(5):833-842. doi:10.1016/j.amepre.2016.06.013.
28. Borghouts J, Eikey E, Mark G, De Leon C, Schueller SM, Schneider M, et al. Barriers to and facilitators of user engagement with digital mental health interventions: Systematic review. Journal of Medical Internet Research. 2021;23(3):e24387. doi:10.2196/24387.
29. Michie S, Yardley L, West R, Patrick K, Greaves F. Developing and evaluating digital interventions to promote behavior change in health and health care: Recommendations resulting from an international workshop. Journal of Medical Internet Research. 2017;19(6):e232. doi:10.2196/jmir.7126.
30. Victorian State Government. Human-centred design playbook. 2020. Available from: https://www.vic.gov.au/human-centred-design-playbook. Accessed August 12, 2025.
31. Mohr DC, Cuijpers P, Lehman K. Supportive accountability: A model for providing human support to enhance adherence to eHealth interventions. Journal of Medical Internet Research. 2011;13(1):e1602. doi:10.2196/jmir.1602.
32. The Point of Care Foundation. EBCD: Experience-based codesign toolkit. 2023. Available from: https://www.pointofcarefoundation.org.uk/resource/experience-based-codesign-ebcd-toolkit/. Accessed August 12, 2025.
33. Linardon J, Fuller-Tyszkiewicz M. Attrition and adherence in smartphone-delivered interventions for mental health problems: A systematic and meta-analytic review. Journal of Consulting and Clinical Psychology. 2020;88(1):1-13. doi:10.1037/ccp0000450.
34. Linardon J, Cuijpers P, Carlbring P, Messer M, Fuller-Tyszkiewicz M. The efficacy of app-supported smartphone interventions for mental health problems: A meta-analysis of randomized controlled trials. World Psychiatry. 2019;18(3):325-336. doi:10.1002/wps.20673.

35. World Health Organization. Monitoring and evaluating digital health interventions: A practical guide to conducting research and assessment. Geneva: World Health Organization; 2016. Available from: https://www.who.int/publications/i/item/9789241511766. Accessed August 12, 2025.
36. Altman M, Huang TTK, Breland JY. Design thinking in health care. Preventing Chronic Disease. 2018;15:E117. doi:10.5888/pcd15.180128.
37. Braun V, Clarke V. Using thematic analysis in psychology. Qualitative Research in Psychology. 2006;3(2):77-101. doi:10.1191/1478088706qp063oa.
